# Prospective observational study validating the German version of the Control of Allergic Rhinitis and Asthma Test (CARAT10)

**DOI:** 10.1038/s41533-018-0112-8

**Published:** 2018-12-04

**Authors:** Christoph Ulrich Werner, Luisa Koch, Klaus Linde, Levente Kriston, Konrad Schultz, Oxana Atmann, Antonius Schneider

**Affiliations:** 1 Technical University of Munich, TUM School of Medicine, Institute of General Practice, Munich, Germany; 2 University Medical Center Hamburg-Eppendorf, Department of Medical Psychology, Hamburg, Germany; 3 Klinik Bad Reichenhall, Center for Rehabilitation, Pulmonology and Orthopedics, Bad Reichenhall, Germany

## Abstract

The Control of Allergic Rhinitis and Asthma Test (CARAT10), developed by Portuguese experts, is the only questionnaire assessing asthma control under additional consideration of a frequently concurrent allergic rhinitis (AR), providing sub-scores for both diseases. Aims of this study were the translation and validation of its German version. Asthma patients both with and without AR were included at three primary care pulmologists´ practices in Southern Germany. After translation process, patients completed the CARAT10, the Asthma Control Questionnaire (ACQ), the Asthma Control Test (ACT), and the Standardised Asthma Quality of Life Questionnaire (AQLQ(S)). Item and scale characteristics as well as measures of reliability and validity of the CARAT10 were determined. A confirmatory factor analysis was conducted to test factorial validity. Data of 213 patients were analysed, 101 (47%) of them with concurrent AR. Missing responses were minimal and unsystematic. Cronbach´s α was 0.87 for the CARAT10 total score (TS) and 0.84 for each sub-score. Spearman´s correlation coefficients for the association of the CARAT10 TS with ACQ, ACT and AQLQ(S) were moderate to high and slightly higher in patients with AR. Higher correlations were found for its lower airway sub-score than the upper airway sub-score (all around 0.8 to all around 0.3). Confirmatory factor analysis confirmed the two-factorial scale structure of the CARAT10, with a two-factor model showing a better fit to the data than a one-factor model. The German version of the CARAT10 is an acceptable, reliable and valid tool. Our results suggest a recommended use in asthma patients with AR.

## Allergic rhinitis and asthma: German version of control test verified

A newly-translated German version of a two-factor asthma test shows promise as a reliable tool for assessing disease control. Asthma frequently co-exists with allergic rhinitis (AR), a condition in which allergies cause irritation and inflammation of the nasal passages. To verify asthma control under AR, a Portuguese team developed the Control of Allergic Rhinitis and Asthma Test (CARAT10). Now, Christoph Ulrich Werner at the Technical University of Munich in Germany and co-workers have translated CARAT10 into German and verified its reliability and validity for use in the German population. The team trialed their CARAT10 version with 213 patients, 101 of whom had concurrent AR, and compared the results with three standardised asthma questionnaires. They found that CARAT10 was a better fit to verify disease control for patients with co-existing AR than in such.

## Introduction

Since the focus in treatment of asthma changed from the handling of acute events to achieving a maximum of so-called asthma control, the use of questionnaires for assessing asthma symptom control has gained importance. Assessing asthma control means assessment of the control of the disease, which consists of the control of clinical symptoms (asthma symptom control) as well as the risk for upcoming adverse events.^1^ Available questionnaires mainly measure symptoms, while some of them additionally capture daily activities, health-related quality of life and/or lung function. Widely used questionnaires in assessment of asthma control are the Asthma Control Test (ACT) and the Asthma Control Questionnaire (ACQ), with the ACQ being used in several versions, one of these including lung function.^2^ These questionnaires pay attention to asthma only but not to a possible allergic rhinitis, which often affects asthma control as being associated to asthma as part of the so-called atopic trias: allergic rhinitis, asthma, atopic dermatitis.^3–6^

Therefore, the guidelines of the Allergic Rhinitis and its Impact on Asthma initiative recommend a combined approach for management of both asthma and allergic rhinitis.^7^ As the only questionnaire assessing both diseases at once, the Rhinasthma, focused on the impairment of health-related quality of life, between 2007 and 2009 a group of Portuguese experts developed the Control of Allergic Rhinitis and Asthma Test (CARAT), a questionnaire with 17 questions.^8,9^ Subsequently, they reduced it in a following study to its currently used version, the CARAT10, consisting of 10 items.^10^

This new questionnaire quantifies control of both allergic rhinitis and asthma (ARA) in patients with a previous diagnosis of ARA. It asks for symptoms of the upper and lower airway tract, limitations in daily tasks, waking up at night, and increased use of medications. A previous exploratory factor analysis had shown a two-factorial structure of the CARAT10, suggesting that four items could be summarised in a rhinitis score and six items in an asthma related score.^10^ Therefore, the CARAT10 gives the possibility of assessing both diseases together or separately by providing the three scoring options of a 'Total Score' (TS), a 'Score of the upper airway' (SUA) for AR and a 'Score of the lower airway' (SLA) for asthma.^10^

The questionnaire was developed and validated in Portuguese and since then translated in several other languages, but so far not in German. This study aimed to translate the CARAT10 in German and to investigate the German versions´ reliability, convergent and discriminant validity and factorial validity. Therefore, we estimated its internal consistency (reliability), compared it with other questionnaires used in assessment of asthma control and quality of life (convergent and discriminant validity), and conducted a confirmatory factor analysis (factorial validity).

## Results

### Recruitment of participants

Between November 2016 and June 2017, 260 patients were invited to participate in this study, of whom 38 could not be included. Three patients did not meet all inclusion criteria and 35 patients were not willing to participate for no special reason. The other 222 patients participated answering their sets of questionnaires and measuring lung function with 92% completing the questionnaires without any missing CARAT10 item. Nine of all participants had to be excluded from analysis because of more than one CARAT10 item missing in their questionnaires. Finally, 213 patients were included in the psychometric analysis, all of them of Caucasian ethnicity without prior selection.

### Description of study participants

All 213 patients had been diagnosed with bronchial asthma by a specialist prior to the study, 101 (47%) of them with concomitant AR. At the day of inclusion to the study, 119 participants (56%) by specialist´s assessment were classified as currently symptomatic with their asthma and 17 (8%) as currently symptomatic with their AR. On average, patients were 50 years old (SD 16, median 50) with 65% being female. Mean BMI of study participants was 26.4 kg/m^2^, 31 persons (15%) were smokers and 82 (39%) had undergone a patient education programme prior to the study. Of the participating patients 91 (43%) were enrolled in the Disease Management Program (DMP) Asthma of the Bavarian Association of Statutory Health Insurance Physicians. Asthma specific medication was taken by 200 of the 213 (87%), an additional medication against allergic problems by 25 (12%). Characteristics of study participants are shown in Table 1.

**Table 1:** Characteristics of study participants

	Total	Allergic rhinitis	
		Yes	No
Number of participants	213	101 (47.4%)	112 (52.6%)
Age mean (SD) years	50 (16.3)	44.8 (14.6)	54.7 (16.4)
Size mean (SD) cm	170 (9.2)	171 (9.6)	169 (8.9)
BMI mean (SD) kg/m^2^	26.4 (6.1)	26 (6.3)	26.8 (5.9)
Gender *n* (% of total)			
Female	139 (65.3)	63 (45.3)	76 (54.7)
Male	74 (34.7)	38 (51.5)	36 (48.5)
Participant DMP *n* (%)	91 (42.7)	43 (47.3)	48 (52.7)
Smokers/Ex-smokers (%)	31 (14.6)	12 (38.7)	19 (61.3)
Currently symptomatic asthma (%)	119 (55.9)	57 (47.9)	62 (52.1)
Currently symptomatic AR (%)	17 (8)	17 (100)	0 (0)
Previous asthma training (%)	82 (38.5)	41 (50)	41 (50)
Previous asthma medication (%)	200 (87)	94 (47)	106 (53)
Previous anti-allergic medication (%)	25 (11.7)	17 (68)	8 (32)
ACT score mean (SD)	19.7 (4.8)	20.1 (4.9)	19.3 (4.7)
≥20 *n* (%)	129 (62.3)	65 (50.4)	64 (49.6)
<20 *n* (%)	78 (37.7)	34 (43.6)	44 (56.4)
ACQ7-FEV1 score mean (SD)	1.3 (1.0)	1.2 (1.1)	1.4 (1.0)
0–0.75 *n* (%)	81 (38.8)	43 (53.1)	38 (46.9)
0.76–1.49 *n* (%)	54 (25.8)	24 (44.4)	30 (55.6)
≥1.5 *n* (%)	74 (35.4)	32 (43.3)	42 (56.7)
ACQ6 score mean (SD)	1.3 (1.1)	1.2 (1.1)	1.3 (1.1)
ACQ5 score mean (SD)	1.4 (1.2)	1.3 (1.3)	1.4 (1.2)
CARAT10			
Total score mean (SD)	19.7 (7.1)	18.4 (7.6)	20.8 (6.7)
>24 *n* (%)	67 (31.5)	25 (37.3)	42 (62.7)
≤24 *n* (%)	146 (68.5)	76 (52.1)	70 (47.9)
Score of the upper airway mean (SD)	6.5 (3.8)	5.5 (3.8)	7.4 (3.7)
>8 *n* (%)	74 (36.5)	26 (35.1)	48 (64.9)
≤8 *n* (%)	129 (63.5)	72 (55.8)	57 (44.2)
Score of the lower airway mean (SD)	13.2 (4.3)	13.0 (4.7)	13.4 (4.0)
≥16 *n* (%)	78 (36.6)	36 (46.2)	42 (53.8)
<16 *n* (%)	124 (63.4)	56 (45.2)	68 (54.8)
AQLQ total score mean (SD)	5.5 (1.1)	5.6 (1.1)	5.4 (1.1)
FEV1 %predicted mean (SD)	88.6 (17.3)	89.2 (16.9)	88 (17.7)

Numbers are absolute numbers with standard deviations (SD) or percentages (%)

*BMI* body mass index, *DMP* Disease Management Programme, *AR* allergic rhinitis, *ACT* Asthma Control Test, *ACQ* Asthma Control Questionnaire, *FEV1* forced expiratory volume in one second, *CARAT10* Control of Allergic Rhinitis and Asthma Test, *AQLQ* Asthma Quality of Life Questionnaire

### Descriptive item statistics

Responses from 213 participants to the questionnaire were included in the analysis with no sign of single items of the CARAT10 being systematically omitted (maximum three missings per CARAT10 item). Responses for all items covered the full range of answer options (see Table 2). For most items, there was skewness with high score values being more frequent (indicating low symptom load). Corrected item-total correlations were moderate to strong ranging between 0.59 and 0.72, except for the item on use of medication (0.40). Internal consistency was good, with Cronbach´s α being 0.87 for the TS and 0.84 for both the SUA and the SLA, respectively. Consistency tended to be slightly higher among patients suffering from an AR (Crohnbach’s α = 0.89 vs 0.84 for TS, 0.84 vs 0.81 for SUA and 0.88 vs 0.80 for SLA). The mean CARAT10 TS was 19.7 (SD 7.1) with a lower mean score for those patients with AR (18.4 (7.6)) to those without (20.8 (6.7)). Mean score of the ACT was 19.7 (4.8), of the ACQ6 1.3 (1.1), of the ACQ7-FEV1 1.3 (1.0), and of the AQLQ(S) 5.5 (1.1). Distributions of scores were skewed towards asthma control for the CARAT LAS, the ACT and the ACQ (skewness values around 1.0). Skewness was less pronounced (0.54) for the CARAT10 TS and negligible (0.14) for the CARAT UAS.

**Table 2:** Descriptive statistics of CARAT10 items

	Frequency distribution of responses (%)					Mean scores (SD)				
Item	Missing	Never (3)	Up to 2 days per week (2)	More than 2 days per week (1)	Almost every day or every day (0)	Patients with allergic rhinitis	Patients without allergic rhinitis	All patients	Skewness	Item-total correlations
'Score of the upper airway' items										
1 Nasal obstruction	0.9	27.2	26.3	17.4	28.2	1.3 (1.1)	1.8 (1.2)	1.5 (1.2)	−0.10	0.59
2 Sneezing	1.4	21.6	29.6	17.8	29.6	1.2 (1.1)	1.6 (1.1)	1.4 (1.1)	−0.01	0.74
3 Nasal pruritus	1.4	40.8	19.3	17.4	21.1	1.6 (1.2)	2.0 (1.1)	1.8 (1.2)	−0.40	0.68
4 Rhinorrhoea	0.9	36.2	21.6	18.3	23.0	1.4 (1.2)	2.0 (1.2)	1.7 (1.2)	−0.29	0.66
'Score of the lower airway' items										
5 Dyspnoea	1.4	33.3	34.2	17.4	13.6	2.0 (1.0)	1.8 (1.1)	1.9 (1.0)	−0.55	0.72
6 Wheezing	0.9	51.2	29.6	9.9	8.5	2.2 (0.9)	2.3 (1.0)	2.3 (1.0)	−1.13	0.63
7 Chest tightness	0.9	43.2	33.3	13.1	9.4	2.1 (1.0)	2.1 (1.0)	2.1 (1.0)	−0.85	0.70
8 Daily activities limitation	0.5	40.8	28.2	15.5	15.0	1.9 (1.1)	2.0 (1.1)	2.0 (1.1)	−0.64	0.65
9 Waking up at night	0.5	65.3	20.2	7.0	7.0	2.4 (1.0)	2.5 (0.8)	2.5 (0.9)	−1.59	0.62
10 Medication	0.9	70.0	18.8	0.0	10.3	2.4 (1.0)	2.6 (0.8)	2.5 (0.9)	−1.92	0.40

Frequency distributions of responses (%), means with standard deviations (SD), skewness and item-total correlations for the items of the CARAT10 (*n* = 213); range for all items 0–3

### Factorial validity

**Fig. 1:**
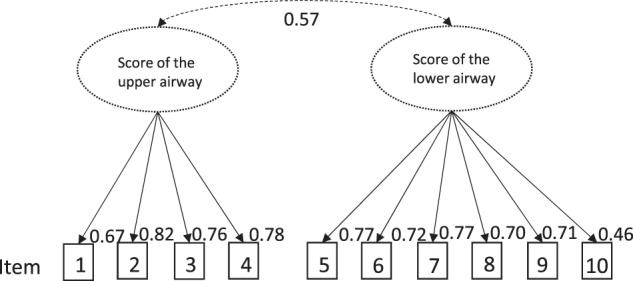
Graph of the confirmatory factor analysis of the two-factor-model—The 10 items of the CARAT10 measure symptoms correlating in an upper airways factor (items 1, 2, 3, 4) and symptoms (items 5, 6, 7), aspects of quality of life (items 8, 9) and use of specific medication (item 10) correlating in a lower airway factor. The broken line arrow indicates correlation between the two factors; continuous arrows indicate factor loads

**Fig. 2:**
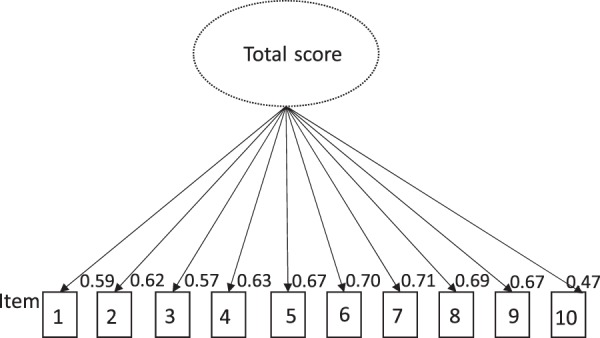
Graph of the confirmatory factor analysis of the one-factor-model—The 10 items of the CARAT10 measure symptoms of the upper airways (items 1, 2, 3, 4), symptoms of the lower airways (items 5, 6, 7), aspects of quality of life (items 8, 9) and use of specific medication (item 10) correlating in an one-factor-total score. The continuous arrows indicate factor loads

Confirmatory factor analysis confirmed the two-factorial structure (Fig. 1). Non-standardised factor loadings were all statistically significant and standardised factor loadings varied between 0.46 and 0.82. The model fit was acceptable (*n* = 191, CFI = 0.95, TLI = 0.93, RMSEA = 0.08 and SRMR = 0.05, standardised chi-square 2.17). The moderate correlation between the two factors (0.57) justifies both the calculation of a total score and sub-scores. The global model fit of the two-factor model was better than the fit of a model assuming one general factor (CFI = 0.75, TLI = 0.67, RMSEA = 0.17, SRMR = 0.10) (Fig. 2).

### Convergent and discriminant validity

Following the results of the confirmatory factor analysis, correlations of all three CARAT10 scores with the ACQ (ACQ6 and ACQ7-FEV1), the ACT and the AQLQ(S) were investigated (see Table 3). In the analysis of all study participants, correlation coefficients were moderate to high for the CARAT10 TS (ranging from 0.60 to 0.71) and high for the SLA (0.78 to 0.87), indicating high convergent validity (these scores are similar to those of well-established instruments for asthma). Correlation coefficients for the SUA were low to moderate (0.18 to 0.40) with not all of them being significant, indicating discriminant validity of SUA and SLA. When analysing patient subgroups with and without an AR, CARAT10 scores and sub-scores showed slightly higher correlations with the other questionnaires in those patients with an AR.

**Table 3:** Spearman´s correlation coefficients between questionnaires and CARAT10 and its subscales

	ACQ5	ACQ6	ACQ7-FEV1	ACT	AQLQ
CARAT10 TS	−0.67**	−0.66**	−0.61**	0.60**	0.71**
CARAT10 TS cAR	−0.76**	−0.75**	−0.71**	0.70**	0.79**
CARAT10 TS sAR	−0.34**	−0.61**	−0.54**	0.55**	0.68**
CARAT10 SUA	−0.31**	−0.31**	−0.25**	0.24**	0.40**
CARAT10 SUA cAR	−0.40**	-0.39**	-0.33**	0.36**	0.48**
CARAT10 SUA sAR	−0.29**	-0.26**	-0.21*	0.18	0.37**
CARAT10 SLA	−0.81**	−0.81**	−0.78**	0.79**	0.87**
CARAT10 SLA cAR	−0.85**	−0.86**	−0.84**	0.84**	0.85**
CARAT10 SLA sAR	−0.77**	−0.76**	−0.72**	0.74**	0.80**

*ACQ* Asthma Control Questionnaire, *FEV1* forced expiratory volume in one second, *ACT* Asthma Control Test, *AQLQ* Asthma Quality of Life Questionnaire, *CARAT10* Control of Allergic Rhinitis and Asthma Test, *TS* total score, *SUA* score of the upper airway, *SLA s*core of the lower airway, *cAR* with allergic rhinitis, *sAR* without allergic rhinitis

**shows a significance of *p* < 0.01; *shows a significance of *p* < 0.05

**Fig. 3:**
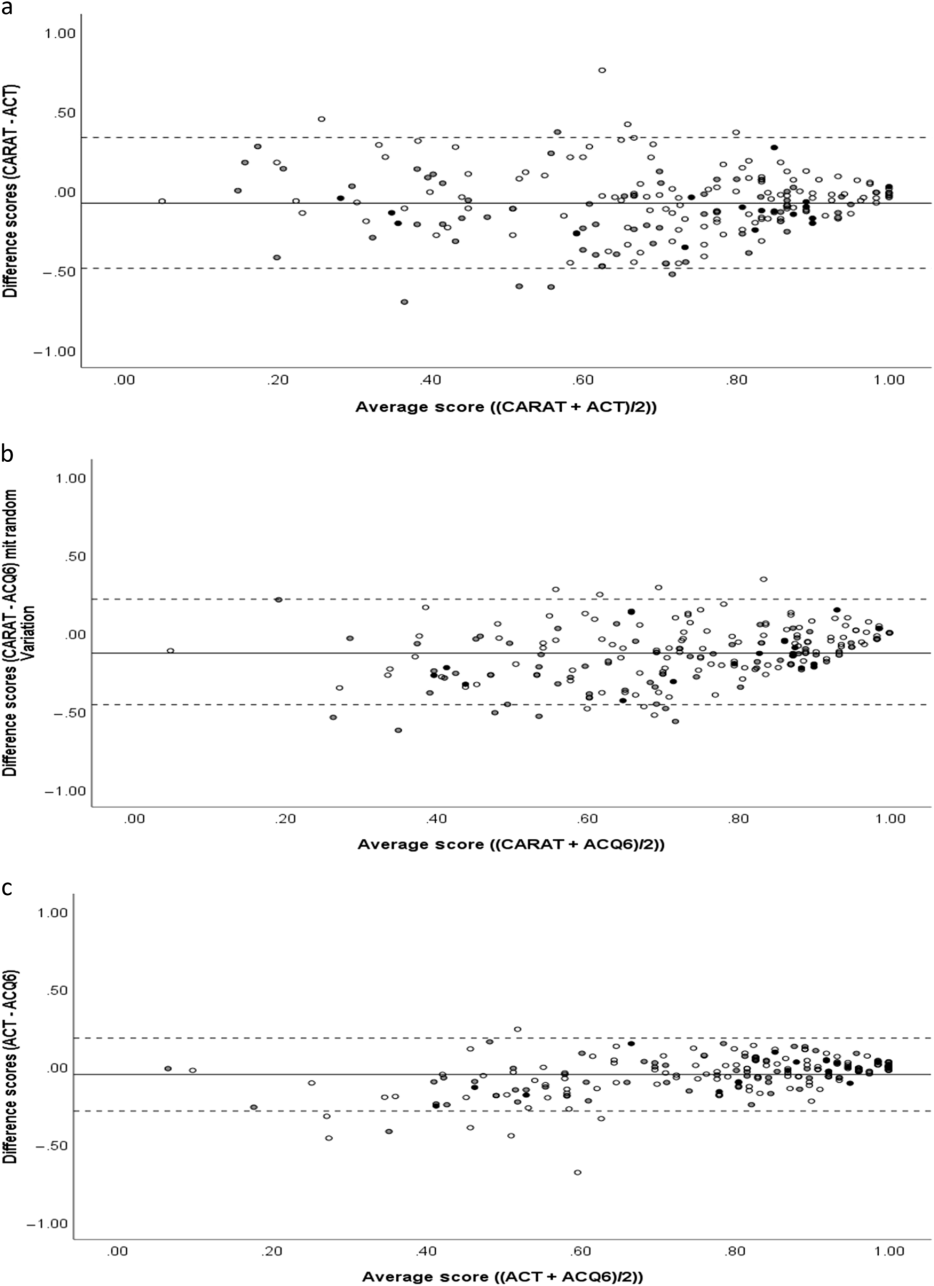
Bland-Altman Plots comparing CARAT10 TS and ACT (upper part), CARAT10 TS and ACQ6 (middle), and between ACT and ACQ6 (lower part). All scales are transformed to a scale ranging from 0 (worst symptom load possible) to 1 (no symptom load). Black filled circles represent value pairs from patients with symptomatic AR, grey filled circles from patients with AR currently not active, and light circles from patients without AR. The solid lines indicates the mean difference between the two scales compared, the dashed lines ± 1.96 standard deviations

Figure 3 depicts Bland-Altman plots comparing the CARAT10 TS, the ACQ6 and the ACT. On average, CARAT10 TS scores tended to be slightly lower (higher symptom burden) than those of the ACQ6 (difference −0.13; *p* < 0.001) and the ACT (−0.08; *p* < 0.001). Compared to the ACT, the ACQ6 also yielded lower score (0.05; *p* < 0.001). Standard deviations were largest for differences between CARAT10 TS and ACT (0.21), slightly smaller between CARAT10 TS and ACQ6 (0.18), and smallest between ACT and ACQ6 (0.12). When the CARAT10 SLA was compared with ACT and ACQ, both differences and standard deviations were smaller (vs. ACT mean -0.01, standard deviation 0.16; vs. ACQ6 mean 0.06, standard deviation 0.14), indicating a higher absolute agreement with the other measures compared to the CARAT10 TS.

## Discussion

The results of our study showed that the German version of the CARAT10 is an acceptable, reliable, and valid instrument. The internal consistency was good, being similar to those of the Portuguese version. The associations with other measures supported convergent validity. The two-factorial structure was confirmed.

The first step of this validation process was the forward/backward translation of the CARAT10. It could be considered a limitation of our study that the German version was translated from the official English version and not from the original Portuguese version. However, the original validation articles of the CARAT10 were published in English. The English version is well known and was used in multiple studies. More than that, during the whole translation process, we were supported by two of the leading Portuguese experts involved in development and validation of the CARAT10.

All patients in our study were treated by an asthma specialist routinely and were used to complete questionnaires, being familiar with the ACT, which is used regularly in the participating practices being promoted by most of German pulmologists. In addition, in all studies about the development of a questionnaire regarding asthma control, participating patients were treated by an asthma specialist before, most of them routinely. This could imply some general bias regarding patient selection. However, only 38.5% of participants underwent a patient education programme prior to the study. This stands against a selection of patients on a higher level of education about asthma and its management above average.

The very high average FEV1 %-predicted value of 88.6% (SD 17.3) in our study could be interpreted as a sign of pre-selection of mainly well-controlled asthma patients, which is refuted by the mean results of the used questionnaires, showing a population of rather uncontrolled patients.

A positive aspect of our study is the low number of missing values with 92% of participants completing the CARAT10 without any missing item value and overall only 113 missing item values (1.0%). These numbers indicate a high acceptance of the translated instrument, especially regarding the fact that the number of participants is even a bit higher than in the original validation study of the CARAT10.^10^

The percentages of participating patients with and without an allergic rhinitis were nearly the same. However, only 8% of patients (17% of those with AR) were symptomatic with their pre-diagnosed AR, and only 12% of participants were taking any medication against an AR, but 56% of participants were symptomatic with their asthma. Therefore, it could be discussed if the influence of AR on asthma control in our study is underrepresented, reducing a potential advantage of the CARAT10 over the other instruments in assessing asthma control.

The CARAT10 and the ACT discriminate only between controlled and uncontrolled patients, while the ACQ also classifies patients in a 'grey zone'.^11,12^ This could influence correlations between questionnaires depending on physician´s focus on patients being controlled or on such uncontrolled. The AQLQ(S) was not developed for nor validated in assessment of asthma control and only one sub-score of it is focused on symptoms like the other questionnaires. Therefore, it mainly measures another dimension or aspect of the control of disease. Its correlations with the CARAT10 would be more interesting if CARAT10´s reliability was investigated when assessing these other aspects.

This study´s results mainly match those of previous studies in development and validation of the CARAT10.^9,10,13,14^ Similar to what was reported in the original development study of the CARAT, patients did not seem to have difficulties understanding the German version of the CARAT10, which supports its feasibility.^9^

The internal consistency was good, with Crohnbach´s alpha overall being similar to those of other studies.^10,13,14^ Our data provided the same Crohnbach´s alpha for the SUA and the SLA of the complete study sample, while the other studies showed different constellations of varying sub-score consistencies. Discriminating patients, there was slightly higher consistency in analysis of those patients suffering from an AR and higher Cronbach’s alpha for the SLA than the SUA in these patients. This meets the results of the two studies of Fonseca et al. All patients in these studies were suffering from ARA, while in the study of van der Leeuw et al. there is a smaller and very heterogeneous sample of patients with only few patients suffering from both diseases. Regarding this and the differing results between patients with or without an AR in our study, these consistencies can be understood as an indication for a preferred use of the CARAT10 in patients with both conditions.

Mean scores of CARAT10 were on quite the same level or just slightly higher than in previous studies. While correlations (relative agreement) of the CARAT10 TS with ACQ5 and ACT in our study population were similar to those of previous studies, correlation coefficients for subscales tended to differ. Correlation coefficients for the SUA in our unselected study sample were low to moderate (0.24 to 0.40), contrary to those of the CARAT10 TS and SLA, indicating that this scale seems to measures a different dimension. Bland-Altman analyses suggested that the CARAT10 does not show a full absolute agreement with the ACT and ACQ. This implies that some upward correction of the CARAT10 scores is necessary, if comparing scores directly, which are obtained by different measures.

When discriminating patients into those with both asthma and AR, and those with asthma only, correlations of all three CARAT10 scores with ACQ, ACT and AQLQ(S) consistently were slightly lower in the latter group. Correlations of the CARAT10 SUA in the group without AR were of weaker significance and partly non-significant (see Table 3). This suggests that the co-existence of AR has an impact on the rating of asthma symptoms. However, for practical reasons, the ensuing and still ongoing analysis of the collected data including the results of gathered specialist ratings and lung function parameters investigating a possibly different usefulness of the CARAT10 in asthma patients with and without an AR will be presented in a following article.

The confirmatory factor analysis confirmed the two-factorial structure, contrary to structures of the ACQ and the ACT.^15^ It showed a better model global fit for the two-factorial model than for the model with only one factor. The correlation between the two factors was around 0.6. This suggests that the two factors measure two related but not identical aspects of airway disease control. This concurs with the fact that asthma and AR are two of the spectrum of allergic diseases, both being part of the atopic trias. On the one hand, this could lead to the conclusion that the subscales would better be used for the specific diseases separated from each other. On the other hand, it could indicate that the CARAT10 questionnaire and its TS preferably should be used in patients with both AR and asthma.

In conclusion, the results of our study confirm acceptability, reliability, validity, and internal consistency of the German version of the CARAT10. However, our results suggest a possibly higher usefulness of the CARAT10 in asthma patients with AR than in those without.

## Methods

### Study design

The study was a non-interventional prospective observational study. It was approved by the Ethics Committee of the Medical Faculty of the Technical University Munich (#327/16 S). All patients gave written informed consent to their participation.

### Patient recruitment and inclusion criteria

At three private pulmologist practices in Southern Germany, patients with confirmed asthma diagnosis visiting the practices regularly were informed about the study and invited to participate during a routine office visit. Their asthma control was assessed by 10 different pulmologists working in these practices. Inclusion criteria were age ≥ 18 years, asthma diagnosis confirmed via spirometry or bronchial provocation, sufficient knowledge of German language, regular control consultations because of asthma, and a signed declaration of informed consent. Patients were offered no incentives or other benefits.

### Questionnaires

#### CARAT10

First, we translated the CARAT10 into German. The translation of the official English version (taken from the official website: www.caratnetwork.org) into German followed the three main steps of the official translation protocol.^16^ The English version was translated into German by two researchers, independently. A German version was created in cooperation with two other researchers and the CARAT team and then translated back into English. After revision by the CARAT team including Portuguese developers of CARAT10 Joao A. Fonseca and Jaime Correia de Sousa an agreed version then was set up. Afterwards, this agreed version was tested in 19 adult asthma patients in a specialised pulmonary rehabilitation centre in Bavaria in March 2016 regarding understanding, wording and interpretation of the questionnaire. Integrating patients´ comments, a final German version of the CARAT10 was set up.

The CARAT10 is a self-administered questionnaire consisting of 10 questions referring to the previous 4 weeks. Every question has the answer options “Never” (three points), 'Up to 2 days per week' (two points), 'More than 2 days per week' (one point) and 'Almost every day or every day' (zero points). Findings are summarised in three scores: the 'Total score' (TS) ranging from 0 to 30 points (>24 indicating good disease control) and two sub-scores, with the 'Score of the upper airway' (SUA) ranging from 0 to 12 (>8 indicating disease control) and the 'Score of the lower airway' (SLA) from 0 to 18 points (≥16 indicating disease control).^17^ The first four questions address symptoms of a rhinitis (blocked nose, sneezing, itchy nose, runny nose) being summarised in the SUA. The following six questions result in the SLA. Three questions concern symptoms of the lower airway tract (shortness of breath/dyspnoea, wheezing in the chest, chest tightness upon physical exercise), and two questions regard aspects of quality of life because of the allergic respiratory disease (tiredness/limitations in doing daily tasks, waking up during the night). One question refers to a possibly increased need for medication.

#### Asthma Control Questionnaire

This self-administered questionnaire comprises five questions regarding asthma symptoms in the previous week (sleeping impairment, severity of symptoms in the morning, limitation in daily activities, frequencies of dyspnoea and wheezing), one question concerns the need for reliever medication and another covers lung function parameters gathered before the use of a bronchodilator.^18^ The response to every question is rated on a 7-point Likert scale, ranging from zero to six, zero meaning absolute control. For lung function, measurements of FEV1 or PEF (FEV1 in this study) are taken and transferred into percentage of nominal value and then into a score from zero (observed values > 95% of predicted value) to six (<50%). By adding all scores for single items and dividing the result by the number of items, summary scores are calculated to define the degree of asthma control. Three different versions of the ACQ can be calculated: the ACQ5, consisting of the five questions about symptoms only, the ACQ6, consisting of ACQ5 (symptoms) and need of reliever medication, and ACQ7, consisting of questions about symptoms, medications and lung function parameters. Asthma control is indicated by the same cut-off points in all three versions according to GINA guidelines (well controlled ≤ 0.75, 'grey zone' 0.76–1.49, ≥1.5 uncontrolled).^12,19^

#### Asthma Control Test

The Asthma Control Test is self-administered and consists of five questions about activity limitations, sleeping impairment, asthma symptoms, need for reliever medication and patients self-rating of asthma control in the previous 4 weeks, each of them being rated from one to five points. A summary score then is calculated ranging from 5 (worst values) to 25 (best values). According to the developers’ advice, an ACT score ≥ 20 was used to identify patients with controlled asthma.^20^

#### Standardised Asthma Quality of Life Questionnaire (AQLQ)

The AQLQ is a tool to assess quality of life of asthma patients by measuring physical, emotional, occupational and social problems that are burdensome to asthma patients.^21,22^ It is available in different versions. We used the self-administered format of the standardised version AQLQ(S) with 32 questions about four aspects of patients´ asthma related quality of life (12 questions about symptoms, 11 about impairments in doing standardised activities, five about emotions and four about environmental influences) regarding the previous 2 weeks. For every question, there are seven response options, which are scored from one to seven, with one being the worst result. A total score can be calculated as well as sub-scores for every of the four sub-aspects by calculating means, but there is no defined cut-off point neither are there units but a Minimal Important Difference, which for all versions of the AQLQ has been established as being close to 0.5.

### Study process

From November 2016 to June 2017, patients with confirmed diagnosis of asthma entering the practice for a previously scheduled routine visit, preferably those participating in the DMP of the Association of Bavarian Statutory Health Insurance Physicians, were informed about the study and offered to participate. If giving written consent, they completed the study questionnaire consisting of the original German versions of the ACQ, the ACT, the AQLQ(S) and the newly translated German version of the CARAT10. After that, practice staff carried out measurements of FEV1 and other lung function parameters by a body plethysmography.

### Statistics

Similar to original validation study of the CARAT10, we aimed to recruit 300 patients to ensure a gathering of at least 100 participants per subgroup (with AR or without).^10^ We did not perform a formal sample size calculation. Data were statistically processed using IBM SPSS Statistics 24 (IBM Corp., Armonk, NY). Standard descriptive statistical techniques (means and standard deviations, absolute and relative frequencies) were used to describe the study sample and basic findings. Differences between patients with and without AR were investigated using Chi-square tests, Student *t*-tests and Mann–Whitney *U*-tests. Means and standard deviations, frequency of missing values, skewness and corrected item-total correlations were computed to reflect psychometric properties of CARAT10 items. Internal consistency was assessed with Cronbach´s alpha. Confirmatory factor analysis was carried out using SPSS AMOS Version 18.0. Based on the study by Fonseca et al., we assumed a two-factor model (an additional analysis was run assuming one general model).^10^ The comparative fit index, the Tucker-Lewis index, the root mean square error of approximation, and the root mean square residual were used to test model fit (SRMR). Convergent validity (relative agreement) was investigated by calculating the correlation between CARAT10 and other questionnaires using Spearman´s correlation coefficient. Based on previous studies, we expected correlation coefficients to be at least moderate (*r* > 0.5) for correlation of CARAT10 TS and CARAT10 SLA with ACQ-versions and moderate to strong with ACT (*r* = 0.5 to 0.7).^10,13,14,17,23^ We expected lower correlations of the CARAT10 SUA with both of the other questionnaires (*r* < 0.5). To further investigate whether CARAT, ACT and ACQ6 result in similar estimates of symptom load (absolute agreement) we produced Bland-Altman Plots.^24^ In a first step, we transformed the scores from the different instruments to have the same scale ranging from 0 (worst symptom load possible) to 1 (no symptom load). Bland-Altman Plots then depict for each individual participant the difference between two transformed scores (for example CARAT—ACT) on the y-axis and the mean of the two measurements divided by two on the x-axis. If the average of all individual differences is close to zero and their standard deviation is small, there is good absolute agreement between the measurements.

## Data Availability

Based on agreement with patients and our ethics committee, only aggregated and anonymized data are available for members of other research groups or journals on reasonable application to the corresponding author. It is not possible to share any independent patient level data.
